# Rapid and sensitive detection of NADPH via mBFP-mediated enhancement of its fluorescence

**DOI:** 10.1371/journal.pone.0212061

**Published:** 2019-02-11

**Authors:** Sung-Hwan You, Ho-Dong Lim, Dae-Eun Cheong, Eung-Sam Kim, Geun-Joong Kim

**Affiliations:** Department of Biological Sciences and Research Center of Ecomimetics, Chonnam National University, Gwangju, Republic of Korea; Universite Paris-Sud, FRANCE

## Abstract

The reduced form of nicotinamide adenine dinucleotide phosphate (NADPH) functions as a reducing agent involved in many biosynthetic and antioxidant reactions in cells. Therefore, a lots of detection or assaying method of this cofactor are developed and used broadly in various research and application fields. These detection or assay tools, however, have often some problems, such as the low sensitivity, susceptibility to environmental interference and time-consuming pretreatment steps, remaining hurdle to successful quantification of NADPH or its derivatives accurately and immediately. Herein, we present a rapid (assay time < 30 s) and sensitive (detection limit < 2 pmol) detection method of NADPH using metagenome-derived blue fluorescent protein (mBFP), a protein capable of significantly enhancing NADPH fluorescence upon binding to this cofactor. Our method takes advantage of the high specificity of mBFP to NADPH and the immediate fluorescence enhancement upon the addition of mBFP to a solution of interest containing NADPH. We can apply this detection scheme to directly quantitative assessment of NADP(H)-dependent enzyme activities in-vitro, and further accessed to quantitative assay of other nicotine amide cofactors, such as NAD+ and NADH, by coupling assay using NAD(H) kinase. Thus, our method enabled us to quantitatively assess the activity of nicotinamide cofactor-associated enzymes in both bacterial and human cell lysates.

## Introduction

Nicotinamide adenine dinucleotide phosphates (NADPH or NADP^+^) are ubiquitous cofactors and act as “electron cabs” in cell metabolism. Since the reduced form (NADPH) serves as an electron donor in diverse biosynthetic pathways, intracellular pools of both NADPH and its oxidized form (NADP^+^) must be regulated to maintain redox homeostasis [1]. These species are co-substrates for physiologically important enzymes such as cytochrome P450s, dehydrogenases, oxidoreductases, and Beayer-Villiger monooxygenases [2–4]. NADPH is also involved in the production of superoxide and nitric oxides as a part of immune responses, in cell signalling, and in the detoxification of drugs or xenobiotics [5, 6]. Thus, the intracellular level of NADPH is a potential biomarker of metabolic dynamics or disorders [7]. Accordingly, the quantification of NADPH is of interest not only for the development of drugs and diagnostic tools but also for the industrial production of valuable metabolites and chemicals based on NADPH-dependent enzymes.

Various methods for NADPH quantification have been developed and some of them are commercially available. These methods are mostly based on the spectroscopic measurement of reducing reagents, such as tetrazolium salts, coupled with NADPH-dependent enzymes [8, 9]. However, these approaches have some drawbacks including low sensitivity, complex pre-treatment steps, long incubation times and chemical instability [10]. Fluorescence assays, based on measurement of the intrinsic fluorescence of NADPH or other artificial substrates, encounter similar hurdles limiting their application [11, 12]. Although other works previously reported the NAD(P)H-dependent florescence enhancement in terms of cofactor specificity to NADPH or high-fold increase in fluorescence by the short-chain dehydrogenase/reductase (SDR) family protein [13–15], no further study on the detailed or optimized assay conditions for NADPH quantification using these SDR members was performed. Therefore, straightforward approaches for the rapid and sensitive detection of NADPH are highly required.

**Fig 1:**
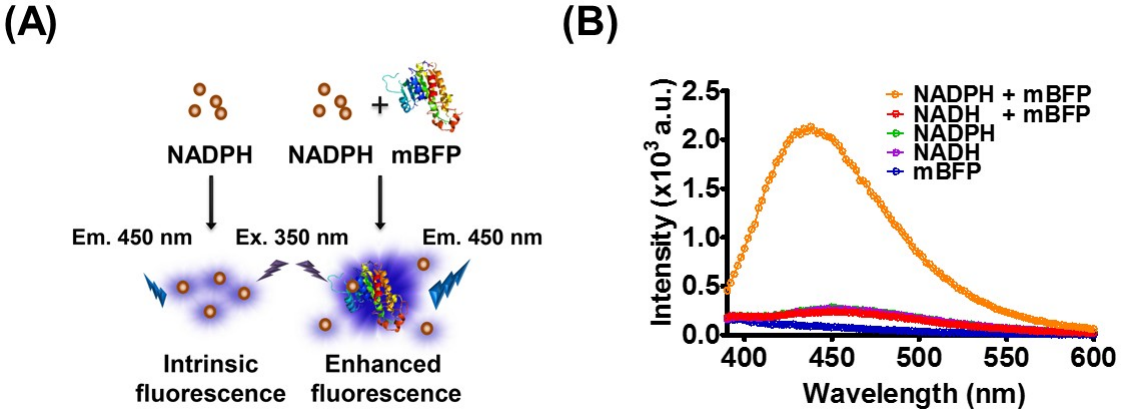
Enhancement of NADPH fluorescence upon its binding to mBFP. (A) Schematic representation of the interaction between NADPH and mBFP followed by immediate enhancement of fluorescence from the complex in comparison with weak fluorescence of free NADPH. (B) Comparison of fluorescence spectra. The emission spectrum between 380 and 600 nm was individually obtained upon irradiation of each solution with UV light at 350 nm wavelength.

We previously isolated and characterized a metagenome-derived blue fluorescent protein (mBFP), a member of the SDR family [16] which efficiently enhanced the intrinsic NADPH fluorescence by ca. 10 times [17], as illustrated in Fig 1A. mBFP was previously believed to be similar to other fluorescent proteins such as GFP or RFP, as it exhibited fluorescence upon ultraviolet (UV) irradiation of mBFP-expressing bacterial cells. It was later demonstrated that the pure protein has no intrinsic fluorescence, but exhibits blue fluorescence in the presence of NADPH [18]. The interaction between mBFP and NADPH resulted in a significant enhancement of this fluorescence signal whereas we observed a weak NADPH-derived fluorescent signal around 450 nm upon excitation at 350 nm (Fig 1B). We also confirmed this result in the context of a study exploring the structural basis of the mBFP/NADPH interaction (manuscript in preparation). These findings led us to hypothesize that mBFP could be employed as a tool to monitor NADPH levels in solutions.

## Materials and methods

### Chemical reagents and fluorescence detection

All chemicals and solvents of reagent grade were purchased from Sigma-Aldrich (St. Louis, MO, USA), unless otherwise specified. Ultra-pure water (18.2 x 10^6^ Ω∙cm) for the preparation of aqueous solutions was supplied by a Milli-Q purification system (Millipore). Two commercially available NADP(H) assay kits were obtained from BioVision (NADP^+^/NADPH Quantification Kit, Cat. No.: K347-100), BioAssay Systems (EnzyChrom^™^ NADP^+^/NADPH Assay Kit, Cat. No.: ECNP-100) and used as controls. mBFP-mediated fluorescence signals were obtained using a 96-well format and a microplate reader (Infinite M200, TECAN, Switzerland) with excitation at 350 nm and emission at 450 nm.

### Plasmids and bacterial strains for protein expression

The expression vector pQE30 (QIAGEN, USA) was used to overexpress mBFP (GenBank: ADG46021.1, 248 amino acids) with an N-terminal 6-His tag. The expression vector pET-22b(+) (Merck, Germany) with a C-terminal His-tag was employed to express NAD(H) kinase. All PCR primers used in this work were listed in S1 Table. The amplified PCR products were treated with appropriate restriction enzymes and inserted into the expression vectors. All constructs were confirmed by DNA sequencing (Macrogen, Seoul, Korea). *Escherichia coli* XL1-Blue and BL21(DE3) were selected as host strains to express mBFP and NADH kinase, respectively. All recombinant strains were cultured in LB medium supplemented with 50 μg/ml ampicillin. The expression of NAD(H) kinase was induced with the addition of 0.2 mM isopropyl-β–D-1-thiogalactopyranoside (IPTG) to the medium at optical density of 0.5 to 0.6.

### Purification of mBFP

A single colony transformed with pQE30-mBFP was inoculated into 25 ml LB medium containing 50 μg/ml ampicillin and pre-cultured at 37 °C with 200 rpm shaking. When the optical density of culture broth at an OD_600_ reached ca. 2.0, the culture was reinoculated into 1 liter of LB medium and further cultured for 8 h under the above-described conditions. Then, the cells were harvested by centrifugation at 6,000×g for 5 min at 4 °C. The mBFP with His-tag at its N terminus was overexpressed without the addition of IPTG (the effect of IPTG induction on the expression level of mBFP was negligible). The harvested cells were washed twice with deionized water and resuspended in a buffer consisting of 20 mM Tris-HCl (pH 7.5) and 300 mM NaCl. After rapid freezing with liquid nitrogen and then slow thawing on ice, cells were disrupted by a sonicator (Vibra Cell VC-750, Sonics Materials) for 10 min using the sonication parameters: 2 s ON/8 s OFF pulse at 40% amplitude and 4 °C. The cell lysate was centrifuged at 16,000×g for 30 min. The supernatant was loaded onto a 5 ml column (His-Trap HF column, GE Healthcare) for affinity chromatography at a flow rate of 4 ml/min after appropriate dilution with a binding buffer consisting of 300 mM NaCl and 20 mM Tris-HCl (pH 7.5). The protein-loaded column was completely washed with the binding buffer containing 20 mM imidazole at a flow rate of 4 ml/min to remove unbound proteins and impurities. The bound protein was recovered using the binding buffer containing 250 mM imidazole. Finally, a desalting column (Sephadex G-10, GE Healthcare) was used to remove imidazole and NaCl from the eluted solution. The resulting His-tagged mBFP showed a high purity (> 95%) and a fluorescence level comparable to that of the wild type (i.e. non-tagged) mBFP upon binding to NADPH. The purified mBFP was kept at -20 °C in 50% glycerol solution to maintain its activity for at least 7 weeks (S4 Fig).

### Optimization of the reaction condition for in vitro assay of NADPH

Various analytical conditions including the incubation time, pH and temperature were evaluated at different molar ratios of NADPH and mBFP to maximize the enhancement of NADPH fluorescence. All assays were performed in a 96-well microplate format with a final volume of 0.2 ml per well. All assay components such as cofactors, mBFP, or cell lysates were mixed in 50 mM Tris-HCl (pH 7.5) buffer. The mBFP-containing reaction solutions were preincubated at 25 °C for 10 min prior to adding cofactors to the solution. The fluorescence signal in the reaction solution was detected by the microplate reader as a function of time.

### Spike-and-recovery assessment for the validation of the mBFP-based NADPH assay

*E*. *coli* XL1-blue and *Candida albicans* NUM678 were cultured in LB and YPD media, respectively. Each cell lysate was prepared as described above. Known amounts of NADPH (0, 30, 50, 100 pmoles/well) were added to the cell lysate. The NADPH level in the spiked cell lysate was quantified by the mBFP-mediated assay. The quantification was compared with those obtained by two commercially available assay kits.

### Assessment of enzymatic activity of G6PDH in artificial conditions

Known amounts (0.05, 0.1, 0.25, 0.5, 1 mU) of glucose-6-phosphate dehydrogenase (G6PDH) were added to a 0.2 ml reaction solution containing 50 mM Tris-HCl buffer (pH 7.5), 5 mM glucose-6-phosphate (G6P), 2 mM NADP^+^, 50 mM MgCl_2_, and 10 μM mBFP. One unit of G6PDH activity is defined as the amount of enzyme required for the formation of 1 μmol NADPH per min at pH 7.5 and 30 °C. The temperatures of the fluorometer and microplate were maintained at 30 °C. Theoretically, 1 mU of G6PDH can produce 40 nmol of NADPH from 400 nmol of NADP^+^.

### Assessment of enzymatic activity of G6PDH in cell lysates

The cultured cells of *E*. *coli*, *Salmonella typhimurium* (NCTC ID: 13347), *Bacillus subtilis* (ATCC ID: 9372), and *C*. *albicans* were harvested and suspended as described above. Cells were disrupted by either treatment with Triton X-100 or sonication. The addition of 0.2% (v/v) Triton X-100 to the resuspended cells was followed by incubation at 30 °C for 10 min. The activity of G6PDH in the chemically disrupted cells was monitored indirectly by measuring NADPH fluorescence upon addition of all the above-described components. In case of physical disruption, the same amount of cells used for chemical disruption were sonicated and centrifuged to obtain a clear supernatant, according to the same procedure used for protein purification, and the enzyme activity was assayed under the same conditions described for chemically-disrupted cells. After incubation of all samples for 30 min, mBFP-mediated fluorescence was measured by the microplate reader. When required, the disrupted cells were desalted by using a dialysis tube (SnakeSkin, Thermo Fisher Scientific) to remove intracellular cofactors and metabolites.

### Activity assay of NAD(H) kinase using mBFP

The assay format for quantitative determination of other cofactors such as NAD^+^ and NADH was established by coupled enzyme reactions using NAD(H) kinase and G6PDH. The gene encoding NADH kinase (Mfnk, Genbank No: AB070351.1) was obtained by PCR from the genomic DNA of *Micrococcus luteus* (ATCC ID: 10240). The activity of purified kinase was assessed as previously reported [19]. The reaction solution contained 50 mM Tris-HCl buffer (pH 7.5), 0.5 mM NADH, 0.1 mM MgCl_2,_ 0.5 mM ATP, purified MfnK (30 μg/well), and G6PDH (0.5 units). Finally, mBFP (5 μM) was added to the reaction solution and the fluorescence of NADPH resulting from NADH phosphorylation directly or via NAD^+^ phosphorylation followed by G6PDH reaction measured in the microplate format.

### Activity assay of NAD(H)- or NADP(H)-dependent dehydrogenases in human cancer cells using mBFP

To measure the activity of NAD^+^-or NADP^+^-dependent enzymes in mammalian cells, MDA-MB-453 (ATCC ID: HTB-131), a human breast cancer cell line was cultured in Dulbecco’s Modified Eagle Medium (DMEM) containing 10% fetal bovine serum (FBS), 100 units/ml penicillin, 0.1 mg/ml streptomycin, and 0.25 μg/ml amphotericin B at 37 °C in a 5% CO_2_ atmosphere. Cells were harvested with Trypsin-EDTA solution and washed twice with phosphate-buffered saline. After resuspension, cells were sonicated and centrifuged at 10,000×g and 4 °C for 20 min and the supernatant was recovered. Four different substrates including G6P, lactate, isocitrate, and malate, all at the final concentration of 2 mM, were separately added to the reaction solution containing 0.2 μg of soluble proteins from cancer cells. The reaction solution for NAD(H)-dependent enzymes contained 1 mM ATP, 0.2 mM MgCl_2_, 3 μM NADH kinase, 0.5 mM NAD^+^, and 10 μM mBFP, while the reaction solution for NADP(H)-dependent enzymes included 0.5 mM NADP^+^ and 10 μM mBFP.

### Statistics

All the experiments were carried out at least three independent times and the quantitative results were expressed as mean ± S.D. Data were compared using Student’s *t*-test. Statistical significance was set at *p*<0.05.

## Results and discussion

**Fig 2:**
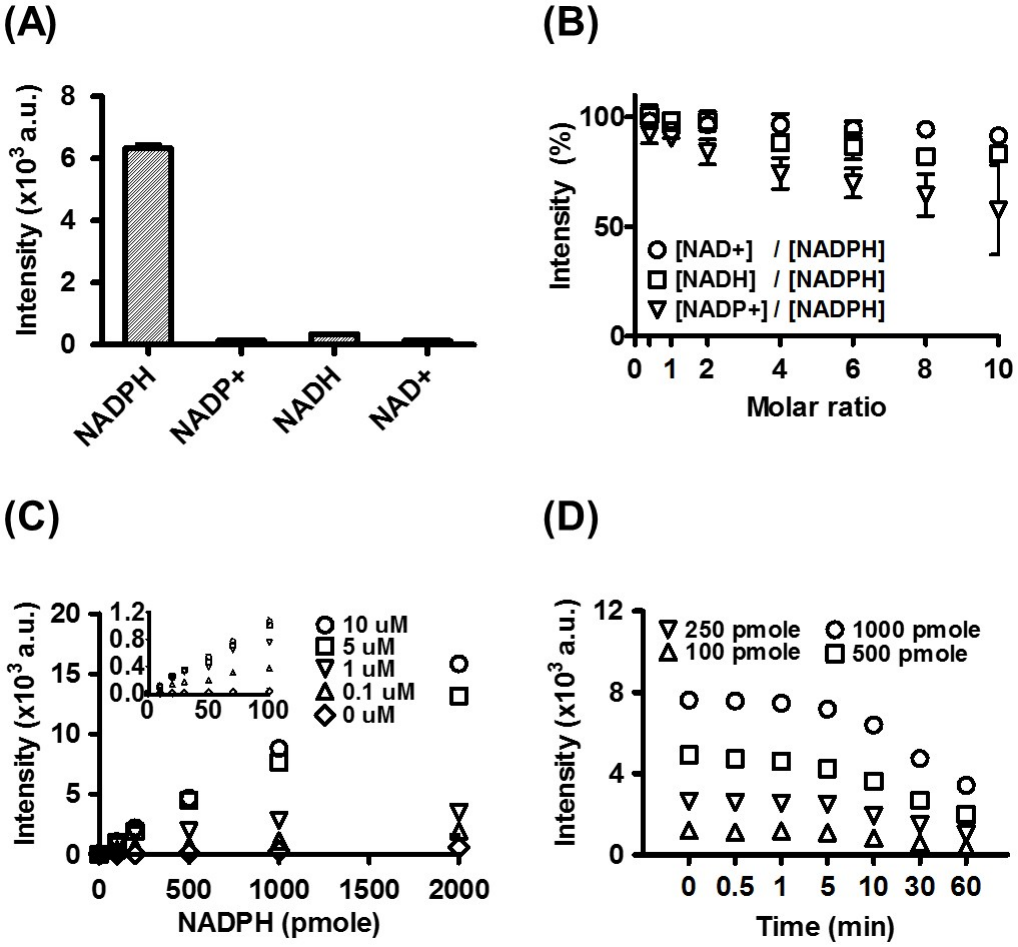
Characterization of mBFP-mediated fluorescence enhancement. (A) Impact of mBFP on different nicotinamide cofactors. (B) Effect of non-NADPH nicotinamide cofactors on the fluorescence level of mBFP-NADPH complexes. (C) Dependency of fluorescence signal on the quantity of NADPH (pmol/200 μl in each well) upon the addition of different concentrations of mBFP. (D) Stability of fluorescent signals after the addition of mBFP to solutions containing different amounts of NADPH. Note that the error bars may not be visible if their sizes are smaller than those of symbols.

To test our hypothesis, we first optimized the assay conditions for detection of NADPH in solution. A recombinant mBFP with a 6xHis tag was expressed in *E*. *coli* XL1-blue. Protein purification from the crude extract by affinity chromatography resulted in highly pure mBFP (>95%). An immediate enhancement of fluorescence was detected when the purified mBFP was added to the NADPH-containing solution, whereas mBFP addition to other nicotinamide cofactors produced negligible changes (Fig 2A), even at concentrations of NAD^+^ or NADH higher than NADPH. However, excess NADP^+^ resulted in competition for mBFP binding and attenuated the NADPH fluorescence enhancement (Fig 2B). When a fixed amount of mBFP was used with increasing amounts of NAPDH, a linear increment in fluorescence was observed up to 2,000 pmol of NADPH per microplate well (Fig 2C). The addition of 5 μM and 10 μM mBFP resulted in linear profiles with correlation coefficients of 0.990 and 0.996, respectively. Remarkably, in the presence of 10 μM mBFP, as little as 2 pmol of NADPH could be detected. When smaller amounts of mBFP were used, the linearity was reduced. The mBFP-induced fluorescence signal was found to decrease over time (Fig 2D). Signal intensity dropped to ca. 50% of the initial value after 1 hour of mBFP addition. However, the initial intensity was nearly unchanged during the first 5 min. A higher increase in fluorescence was obtained at pH 7.5 than with acidic or basic solutions (S1A Fig). Although low temperatures resulted in higher increases in fluorescence (S1B Fig), we decided to perform all assays at 25 to 30°C to better preserve enzymatic activity.

Thus, optimal assay conditions for NADPH quantification included sample preparation at pH 7.5, use of mBFP at the final concentration of 10 μM, and measurement of mBFP-induced NADPH fluorescence at room temperature, within the first 5 min of incubation. For each sample, a single round of detection (addition of mBFP and acquisition of fluorescence) was usually completed within 30 s. Notably, the optimal incubation temperature may depend on the purpose of the assay. If NADPH quantification is the final aim, low-temperature incubation is preferable. On the other hand, for enzyme kinetic profiles where NADPH is produced or consumed, the incubation temperature should be higher than room temperature, for optimal enzymatic activity.

To evaluate the accuracy of mBFP-mediated NADPH quantification, we performed a spike-and-recovery assessment using crude cell lysates of *E*. *coli* and *C*. *albicans*. Two commercially available kits were employed as controls. Our assay showed high accuracy and resulted in 95 to 99% recovery, while for the control kits the recovery was between 70 and 99% (S2 Table: Control A (98 to 99%), Control B (70 to 99%)). The mBFP-mediated assay allowed for rapid and accurate measurement of NADPH spiked into cell lysates and avoided the long incubations typically required by the colorimetric assays.

**Fig 3:**
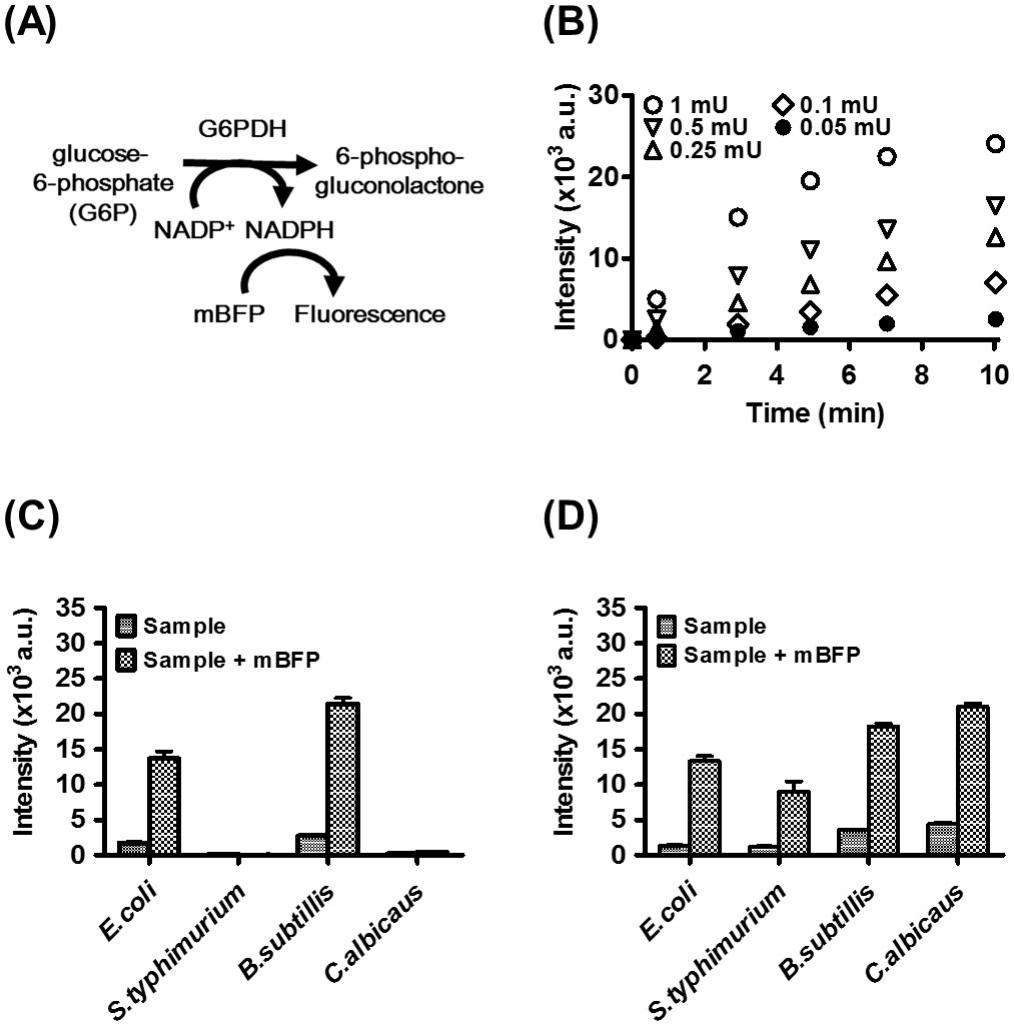
Detection of the activity of NADPH-producing G6PDH. (A) Schematic of NADPH generation from conversion of G6P to 6-phospho-gluconolactone by G6PDH. (B) Kinetic profiles of NADPH production by G6PDH in a microplate format. (C) and (D) Measurement of endogenous G6PDH activity in cell lysates of *E*. *coli*, *S*. *typhimurium*, *B*. *subtilis*, and *C*. *albicans*, after chemical and physical lysis of cells, respectively.

Next, we examined the activity of NADPH-producing glucose-6-phosphate dehydrogenase (G6PDH) using mBFP. G6PDH is an NADP^+^-dependent enzyme that catalyses the conversion of glucose-6-phosphate (G6P) to 6-phospho-gluconolactone in the pentose phosphate pathway, concurrently converting NADP^+^ into NADPH (Fig 3A). The enzymatic production of NADPH was monitored in terms of fluorescence intensity over time, in the presence of different amounts of G6PDH (Fig 3B). Newly produced NADPH resulted in a raise of mBFP-mediated fluorescence. High concentrations of G6PDH resulted in faster and more pronounced mBFP-induced raises in fluorescence. Since, under these conditions, NADP^+^ was much more abundant than the newly formed NADPH, the increase in fluorescence due to formation of the mBFP-NADPH complex might be lower than expected. However, it is known that in most cells, under physiological conditions, NADPH is at least ten times more abundant than NADP^+^ [20, 21], which minimizes the competition effect. We employed this assay to compare G6PDH activity in different cell lysates, obtained by either chemical (Fig 3C) or physical (Fig 3D) disruption of the cell wall and membrane. Addition of G6P and NADP^+^ to cell lysates resulted in the production of NADPH by the endogenous G6PDH enzymes and mBFP binding to NAPDH. Interestingly, whereas chemical disruption was sufficient to detect G6PDH activity in *E*. *coli* and *B*. *subtilis* (Fig 3C), the same method resulted in negligible detection of enzymatic activity in *S*. *typhimurium* and *C*. *albicans*. On the other hand, when cells were physically disintegrated, G6PDH-induced mBFP-dependent fluorescence was detectable at significant levels in all cell lines (Fig 3D). The higher enzymatic performance of chemically disrupted *E*. *coli* and *B*. *subtilis* might be partly attributed to the presence of Triton X-100 in these cell lysates, as the detergent augmented the fluorescence of the mBFP-NADPH complex by ca. 30% (S2 Fig).

**Fig 4:**
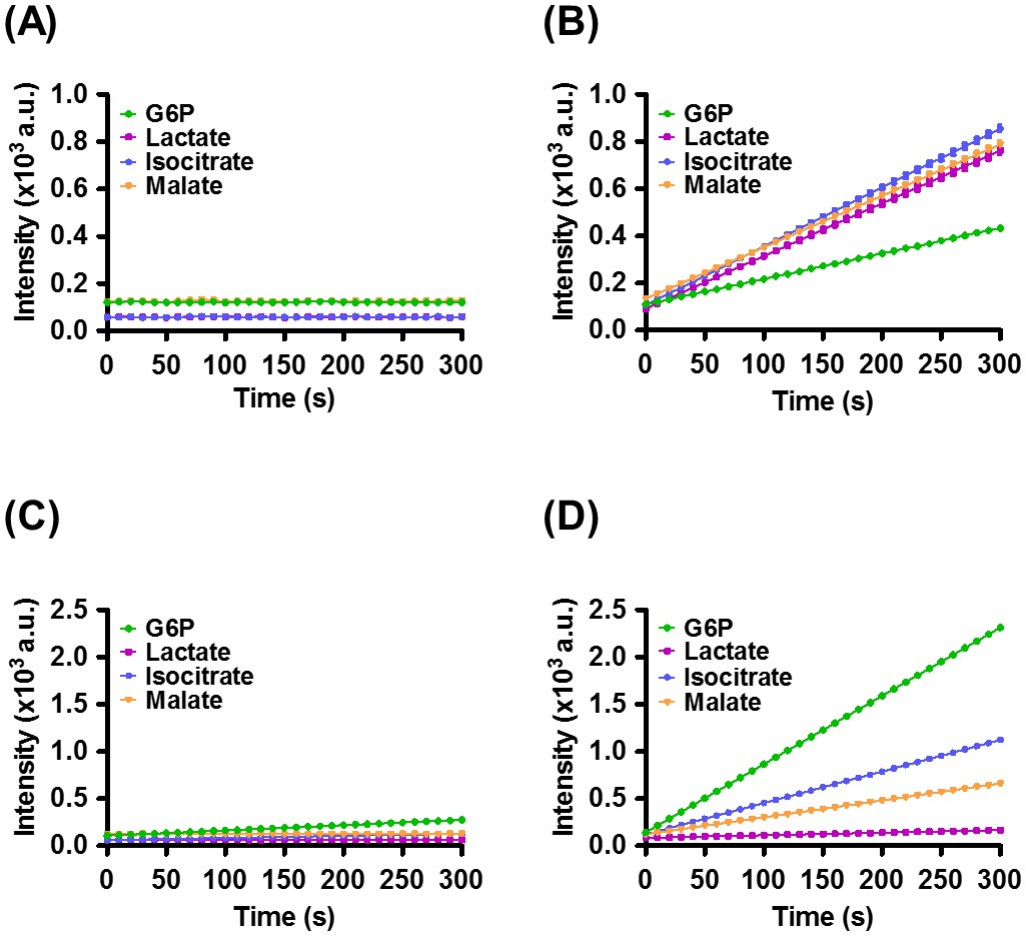
Kinetic profiles of nicotinamide cofactor-associated enzymes in human breast cancer cells. Four substrates including G6P, lactate, isocitrate, and malate were individually added to cell lysates. NAD^+^ and NADP^+^ were employed in the reactions involving NADH- and NADPH-dependent dehydrogenases, respectively. MfnK is an NAD(H) kinase that can phosphorylate NAD(H) into NADP(H) in the presence of ATP. Real-time measurements of fluorescence signals were carried out in four different solutions containing (A) NAD^+^, ATP, and MfnK, (B) NAD^+^, ATP, MfnK, and mBFP, (C) NADP^+^, and (D) NADP^+^ and mBFP.

Finally, we monitored the activity of nicotinamide cofactor-associated enzymes in mammalian cells using mBFP (Fig 4). When glucose is degraded in cells, nicotinamide cofactors such as NAD(H) and NADP(H) are generated or consumed by enzymes involved in glycolysis or TCA cycle, for instance, G6PDH, lactate dehydrogenase (LDH), isocitrate dehydrogenase (IDH), and malate dehydrogenase (MDH) [7, 22, 23]. A cell lysate was obtained from the human breast cancer cell line, MDA-MB-453. The oxidized cofactors, NAD^+^ and NADP^+^, were added to the supernatant and their conversion into NADH and NADPH, respectively, was assessed. An NAD(H) kinase, MfnK [19] was employed for NAD(H) phosphorylation and NADP(H) formation in the presence of ATP (S3 Fig). Individual addition of G6P, lactate, isocitrate, and malate to the lysate promoted the production of NADH or NADPH by the relevant, endogenously expressed enzymes. A small-volume supernatant containing 0.2 μg of soluble proteins was used in these assays to obtain relatively linear kinetic profiles for the four substrates. NADPH production was rapidly detected in the presence of mBFP (Fig 4B and 4D), while a marginal increase in the fluorescence signal was observed in the absence of mBFP (Fig 4A and 4C). In the presence of MfnK, the kinetic profiles of lactate, isocitrate, and malate were characterized by higher fluorescence levels compared to that of the G6P profile because the NADH deriving from oxidation of these substrates by LDH, IDH, and MDH, supplied an extra source of NADPH, resulting in further mBFP-induced fluorescence (Fig 4B). Since G6PDH has a higher turnover rate than do the other dehydrogenases [3], its kinetic profile exhibited the highest fluorescence level in MfnK-free assays (Fig 4D). The time-dependent raise in fluorescence observed for isocitrate and malate in the absence of MfnK may reflect their additional role as substrates in NADP^+^-dependent reactions, involving IDH and MDH, respectively. The marginal fluorescence level for lactate indicated that it is a selective substrate of NAD^+^-dependent enzymes and, therefore, has a negligible impact on NADPH level.

## Conclusions

We developed a rapid, sensitive, and reliable method to quantify the production NADPH and monitor the activity of NADP(H)-dependent enzymes in cell lysates, based on mBFP binding to NADPH and subsequent enhancement of NADPH fluorescence. The mBFP-based approach only requires the addition of purified mBFP to the test solutions and results in rapid and specific, mBFP-induced, NADPH fluorescence. Unlike conventional colorimetric assays, extended incubations (30 min or longer) or additional steps for NAD(H) degradation, such as heating at 65°C, are not required. To our knowledge, our straightforward procedure displays a higher performance, in terms of sensitivity (detection limit 2 pmol), assay time (less than 30 s), linear dynamic range (r^2^ >0.95 between 2 and 1000 pmoles) and fidelity (>95% recovery), compared to the commercially available kits for NADPH detection. The scope of mBFP-based NADPH measurement can be expanded to the monitoring of other nicotinamide cofactor-associated enzymes, by the introduction of NAD(H)-dependent kinases, facilitating the rapid high-throughput screening of enzyme modulators. Our assay is expected to contribute to the characterization of cancer-specific metabolic states and to the development of rapid diagnostic tests for diseases such as bacterial infections in blood or urine.

## Supporting information

S1 Fig Effect of pH and temperature on mBFP-mediated enhancement of NADPH fluorescence. (A) Fluorescence levels of mBFP-NADPH complexes in solutions with different pH values from 6.0 to 10.5 at 30 °C. (B) Fluorescence levels of mBFP-NADPH complexes in solutions with different temperatures from 4 to 37 °C at pH 7.5.
(TIF)

S2 Fig Augmented fluorescence of the mBFP-NADPH complex in the presence of Triton X-100 in the assay solution. The final concentration of the detergent was 0.05% (v/v) (n = 3).
(TIF)

S3 Fig Detection of the activity of MfnK, an NAD(H) kinase, using mBFP. (A) A profile of fluorescence signals due to the enzymatic conversion of NADH to NADPH by MfnK as a function of the NADH amount. The conversion scheme is depicted in the inset. (B) MfnK-dependent conversion of NADH to NADPH was rapidly detected upon addition of mBFP to cell lysates.
(TIF)

S4 Fig Evaluation of stability of mBFP for a long-term storage. mBFP-mediated fluorescence measured at defined time points when mBFP was stored at (A) -20 °C and (B) -80 °C in the different glycerol concentration.
(TIF)

S5 Fig Physical maps of recombinant plasmids used mainly in this work. (A) pQE-mBFP. (B) pET22(+)-MfnK.
(TIF)

S1 Table Primer sequences for the construction of a recombinant plasmids. ^a^ The tagged F and R indicate the forward and reverse primers, respectively. ^b^ The restriction endonuclease recognizes the underlined DNA sequence.
(DOC)

S2 Table Evaluation of spike-and-recovery assays of NADPH in cell lysates. ^a^ Each control is one of the commercially available kits described in the Experimental section. ^b^ Recovery (%) of NAPDH = Measured amount / Expected amount x 100.
(DOC)

S3 Table Fluorescence levels of mBFP-NADPH complexes in solutions at different pH values. ^a^ Mean of three repetitions ± standard deviation of the mean.
(DOC)

S4 Table Fluorescence levels of mBFP-NADPH complexes in solutions at different temperatures. ^a^ Mean of three repetitions ± standard deviation of the mean.
(DOC)

S5 Table Effect of non-NADPH nicotinamide cofactors on the fluorescence level of mBFP-NADPH complexes. ^a^ Mean of three repetitions ± standard deviation of the mean.
(DOC)

S6 Table Dependency of fluorescence signal on the quantity of NADPH upon the addition of different concentrations of mBFP. ^a^ Mean of three repetitions ± standard deviation of the mean.
(DOC)

S7 Table Stability of fluorescent signals after the addition of mBFP to solutions containing different amounts of NADPH. ^a^ Mean of three repetitions ± standard deviation of the mean.
(DOC)

S8 Table Kinetic profiles of NADPH production by G6PDH in a microplate format. ^a^ Mean of three repetitions ± standard deviation of the mean.
(DOC)

## References

[1] Spaans SK, Weusthuis RA, van der Oost J, Kengen SWM. NADPH-generating systems in bacteria and archaea. Frontiers in Microbiology. 2015;6 Artn 742 10.3389/fmicb.2015.00742 26284036 PMC4518329

[2] Pandey AV, Fluck CE. NADPH P450 oxidoreductase: Structure, function, and pathology of diseases. Pharmacol Therapeut. 2013;138(2):229–54. 10.1016/j.pharmthera.2013.01.010 23353702

[3] Lewis CA, Parker SJ, Fiske BP, McCloskey D, Gui DY, Green CR, et al Tracing Compartmentalized NADPH Metabolism in the Cytosol and Mitochondria of Mammalian Cells. Mol Cell. 2014;55(2):253–63. 10.1016/j.molcel.2014.05.008 24882210 PMC4106038

[4] Kamerbeek NM, Janssen DB, van Berkel WJH, Fraaije MW. Baeyer-Villiger monooxygenases, an emerging family of flavin-dependent biocatalysts. Adv Synth Catal. 2003;345(6–7):667–78. 10.1002/adsc.200303014

[5] Ying WH. NAD(+)/ NADH and NADP(+)/NADPH in cellular functions and cell death: Regulation and biological consequences. Antioxid Redox Sign. 2008;10(2):179–206. 10.1089/ars.2007.1672 18020963

[6] Segal BH, Grimm MJ, Khan ANH, Han W, Blackwell TS. Regulation of innate immunity by NADPH oxidase. Free Radical Bio Med. 2012;53(1):72–80. 10.1016/j.freeradbiomed.2012.04.022 22583699 PMC3377837

[7] DeBerardinis RJ, Chandel NS. Fundamentals of cancer metabolism. Sci Adv. 2016;2(5). UNSP e1600200 10.1126/sciadv.1600200 27386546 PMC4928883

[8] Kupfer D, Munsell T. A colorimetric method for the quantitative determination of reduced pyridine nucleotides (NADPH and NADH). Anal Biochem. 1968;25(1):10–6. . 4387535 10.1016/0003-2697(68)90075-4

[9] Zhang ZQ, Yu J, Stanton RC. A method for determination of pyridine nucleotides using a single extract. Analytical Biochemistry. 2000;285(1):163–7. 10.1006/abio.2000.4701 10998277

[10] Wu JT, Wu LH, Knight JA. Stability of NADPH: effect of various factors on the kinetics of degradation. Clinical chemistry. 1986;32(2):314–9. . 3943190

[11] Villette S, Pigaglio-Deshayes S, Vever-Bizet C, Validire P, Bourg-Heckly G. Ultraviolet-induced autofluorescence characterization of normal and tumoral esophageal epithelium cells with quantitation of NAD(P)H. Photoch Photobio Sci. 2006;5(5):483–92. 10.1039/b514801d 16685326

[12] Erban T, Poltronieri P, Stara J. A novel microplate-based HPLC-fluorescence assay for determination of NADPH-cytochrome P450 reductase activity. Biomed Chromatogr. 2012;26(9):1062–5. 10.1002/bmc.1747 22120726

[13] Delabar JM, Martin SR, Bayley PM. The binding of NADH and NADPH to bovine-liver glutamate dehydrogenase. Spectroscopic characterisation. Eur J Biochem. 1982;127(2):367–74. Epub 1982/10/01. . 7140774 10.1111/j.1432-1033.1982.tb06881.x

[14] Li B, Lin SX. Fluorescence-energy transfer in human estradiol 17 beta-dehydrogenase-NADPH complex and studies on the coenzyme binding. Eur J Biochem. 1996;235(1–2):180–6. Epub 1996/01/15. . 8631327 10.1111/j.1432-1033.1996.00180.x

[15] Kao TH, Chen Y, Pai CH, Chang MC, Wang AH. Structure of a NADPH-dependent blue fluorescent protein revealed the unique role of Gly176 on the fluorescence enhancement. J Struct Biol. 2011;174(3):485–93. Epub 2011/03/15. 10.1016/j.jsb.2011.02.010 . 21397029

[16] Kavanagh KL, Jornvall H, Persson B, Oppermann U. Medium- and short-chain dehydrogenase/reductase gene and protein families: the SDR superfamily: functional and structural diversity within a family of metabolic and regulatory enzymes. Cell Mol Life Sci. 2008;65(24):3895–906. 10.1007/s00018-008-8588-y . 19011750 PMC2792337

[17] Hwang CS, Choi ES, Han SS, Kim GJ. Screening of a highly soluble and oxygen-independent blue fluorescent protein from metagenome. Biochem Bioph Res Co. 2012;419(4):676–81. 10.1016/j.bbrc.2012.02.075 22382031

[18] Ng CY, Farasat I, Maranas CD, Salis HM. Rational design of a synthetic Entner-Doudoroff pathway for improved and controllable NADPH regeneration. Metab Eng. 2015;29:86–96. Epub 2015/03/15. 10.1016/j.ymben.2015.03.001 . 25769287

[19] Shi F, Kawai S, Mori S, Kono E, Murata K. Identification of ATP-NADH kinase isozymes and their contribution to supply of NADP(H) in Saccharomyces cerevisiae. Febs J. 2005;272(13):3337–49. 10.1111/j.1742-4658.2005.04749.x 15978040

[20] Ogasawara Y, Funakoshi M, Ishii K. Determination of Reduced Nicotinamide Adenine Dinucleotide Phosphate Concentration Using High-Performance Liquid Chromatography with Fluorescence Detection: Ratio of the Reduced Form as a Biomarker of Oxidative Stress. Biol Pharm Bull. 2009;32(11):1819–23. 10.1248/Bpb.32.1819 19881290

[21] Sallin O, Reymond L, Gondrand C, Raith F, Koch B, Johnsson K. Semisynthetic biosensors for mapping cellular concentrations of nicotinamide adenine dinucleotides. Elife. 2018;7 ARTN e32638 10.7554/eLife.32638 29809136 PMC5990361

[22] Reitman ZJ, Yan H. Isocitrate Dehydrogenase 1 and 2 Mutations in Cancer: Alterations at a Crossroads of Cellular Metabolism. J Natl Cancer I. 2010;102(13):932–41. 10.1093/jnci/djq187 20513808 PMC2897878

[23] Fan J, Ye J, Kamphorst JJ, Shlomi T, Thompson CB, Rabinowitz JD. Quantitative flux analysis reveals folate-dependent NADPH production. Nature. 2014;510(7504):298–302. 10.1038/nature13236 . 24805240 PMC4104482

