# Supplementary figures and images for: Rapid and sensitive detection of NADPH via mBFP-mediated enhancement of its fluorescence
Source: PLoS One. 2019 Feb 11;14(2):e0212061. doi: 10.1371/journal.pone.0212061 (PMC6370209; doi:10.1371/journal.pone.0212061)

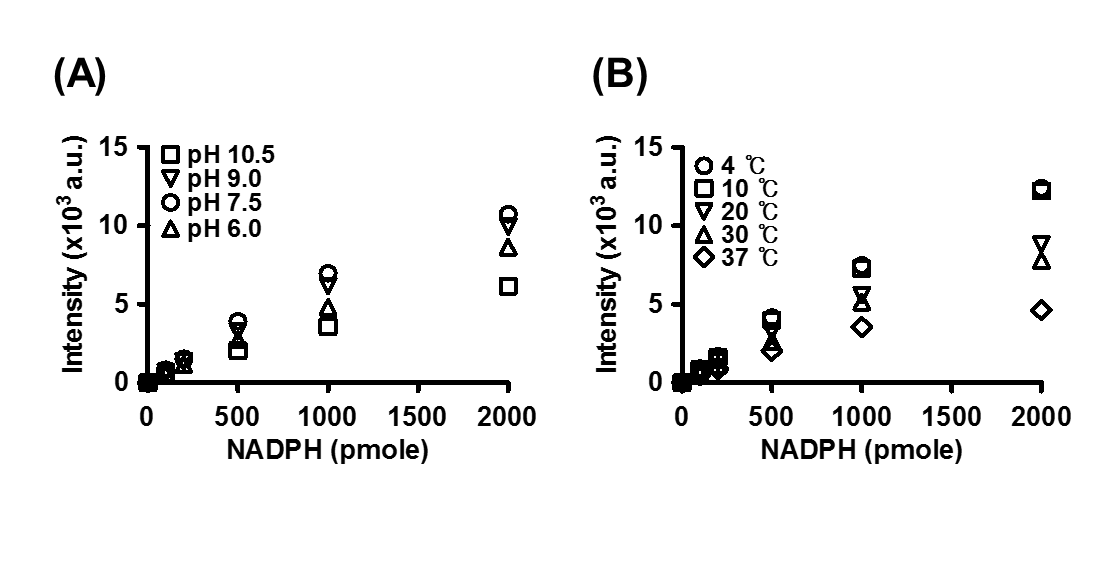

Supplement: S1 Fig — (A) Fluorescence levels of mBFP-NADPH complexes in solutions with different pH values from 6.0 to 10.5 at 30 °C. (B) Fluorescence levels of mBFP-NADPH complexes in solutions with different temperatures from 4 to 37 °C at pH 7.5. (TIF) [file pone.0212061.s001.tif]

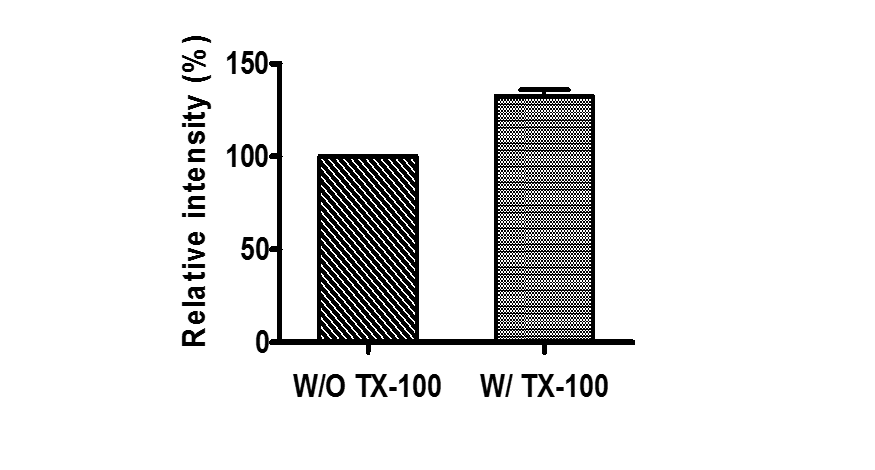

Supplement: S2 Fig — The final concentration of the detergent was 0.05% (v/v) (n = 3). (TIF) [file pone.0212061.s002.tif]

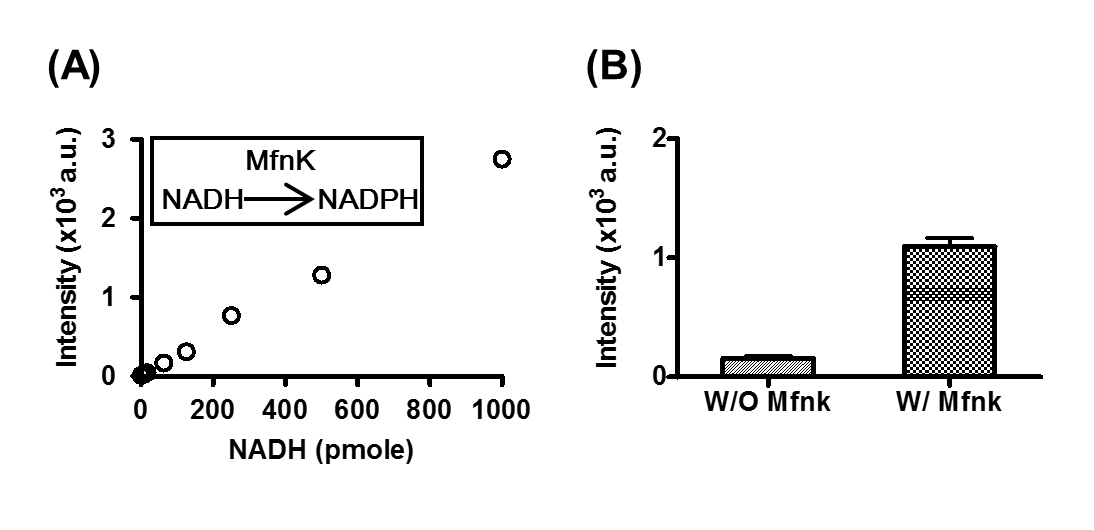

Supplement: S3 Fig — (A) A profile of fluorescence signals due to the enzymatic conversion of NADH to NADPH by MfnK as a function of the NADH amount. The conversion scheme is depicted in the inset. (B) MfnK-dependent conversion of NADH to NADPH was rapidly detected upon addition of mBFP to cell lysates. (TIF) [file pone.0212061.s003.tif]

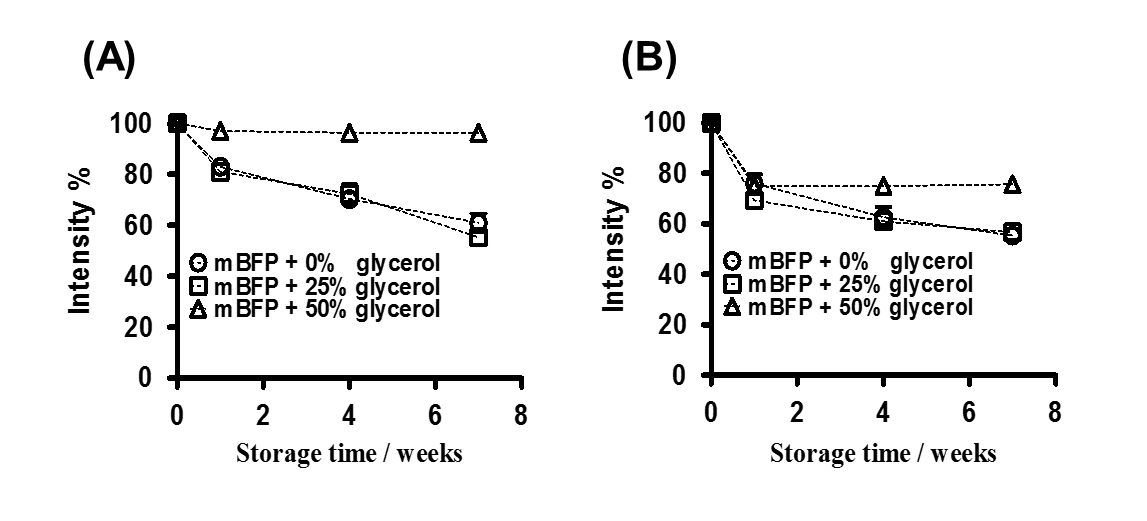

Supplement: S4 Fig — mBFP-mediated fluorescence measured at defined time points when mBFP was stored at (A) -20 °C and (B) -80 °C in the different glycerol concentration. (TIF) [file pone.0212061.s004.tif]

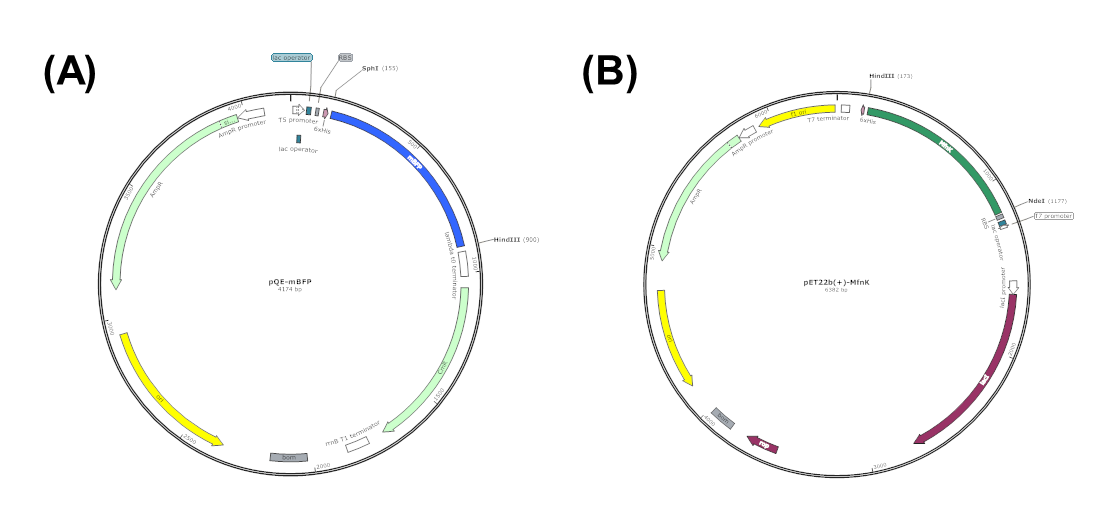

Supplement: S5 Fig — (A) pQE-mBFP. (B) pET22(+)-MfnK. (TIF) [file pone.0212061.s005.tif]
