# Supplementary material for: Rapid and sensitive detection of NADPH via mBFP-mediated enhancement of its fluorescence
Source: PLoS One. 2019 Feb 11;14(2):e0212061. doi: 10.1371/journal.pone.0212061 (PMC6370209; doi:10.1371/journal.pone.0212061)
Supplement: S1 Table — a The tagged F and R indicate the forward and reverse primers, respectively. b The restriction endonuclease recognizes the underlined DNA sequence. (DOC) [file pone.0212061.s006.doc]

# S1 Table. Primer sequences for the construction of a recombinant plasmids

# ^a^ The tagged F and R indicate the forward and reverse primers, respectively.

| Name^a^ | DNA Sequence (5’ 🡪 3’) | RE^b^ site |
| --- | --- | --- |
| QE-mBFP-F | ATAGCATGCCAGAATCTGAACGGCAAAGTGG | *Sph*Ⅰ |
| QE-mBFP-R | ATAAAGCTTTCAAGCGGCGAAGCCG | *Hin*dIII |
| MfnK-F | ATACATATGCCCTACACCCCCGGAC | *Nde*Ⅰ |
| MfnK-R | ATAAAGCTTGTTCGACGACGTCGGGAC | *Hin*dIII |

# ^b^ The restriction endonuclease recognizes the underlined DNA sequence.
