# Supplementary material for: Rapid and sensitive detection of NADPH via mBFP-mediated enhancement of its fluorescence
Source: PLoS One. 2019 Feb 11;14(2):e0212061. doi: 10.1371/journal.pone.0212061 (PMC6370209; doi:10.1371/journal.pone.0212061)
Supplement: S2 Table — a Each control is one of the commercially available kits described in the Experimental section. b Recovery (%) of NAPDH = Measured amount / Expected amount x 100. (DOC) [file pone.0212061.s007.doc]

# S2 Table. Evaluation of spike-and-recovery assays of NADPH in cell lysates

|  |  | mBFP-based assay | | | Control A^a^ | | | Control B^a^ | | |
| --- | --- | --- | --- | --- | --- | --- | --- | --- | --- | --- |
| Cells | Spiked amount ofNADPH(pmol/well) | Measured amount ofNAPDH(pmol/well) | Expectedamount ofNADPH(pmol/well) | Recovery^b^(%) | Measured amount ofNAPDH(pmol/well) | Expectedamount ofNADPH(pmol/well) | Recovery(%) | Measured amount ofNAPDH(pmol/well) | Expectedamount ofNADPH(pmol/well) | Recovery(%) |
| *E.coli* | 0 | 24.5±1.2 | - | - | 21.7±1.1 | - | - | 24.6±0.7 | - | - |
|  | 30 | 52.0±2.9 | 54.5 ± 1.2 | 95.3±3.1 | 51.0± 2.4 | 51.7 ± 1.1 | 98.6±3.6 | 51.2±3.6 | 54.6 ± 0.7 | 93.8±6.9 |
|  | 50 | 70.9±1.2 | 74.5 ± 1.2 | 95.2 ± 3.1 | 71.2±1.9 | 71.7 ± 1.1 | 99.2 ± 2.4 | 71.2±1.6 | 74.6 ± 0.7 | 95.4 ± 1.4 |
|  | 100 | 118.6±4.5 | 124.5 ± 1.2 | 96.6±1.8 | 120.3±1.6 | 121.7 ± 1.1 | 98.8±2.0 | 123.9±2.8 | 124.6 ± 0.7 | 99.4 ± 2.7 |
| *C. albicans* | 0 | 8.3±1.1 | - | - | 8.5 ± 0.8 | - | - | 21.5 ± 4.0 | - | - |
|  | 30 | 37.1 ± 1.7 | 38.3 ± 1.1 | 96.9 ± 5.2 | 37.7 ± 1.0 | 38.8 ± 0.8 | 98.0 ± 1.7 | 35.6 ± 1.2 | 51.0 ± 3.6 | 70.1 ± 4.6 |
|  | 50 | 57.1 ± 2.9 | 58.3 ± 1.1 | 98.0 ± 3.3 | 57.6 ± 1.2 | 58.5 ± 0.8 | 98.5 ± 2.2 | 57.8 ± 1.4 | 71.0 ± 3.6 | 81.6 ± 5.8 |
|  | 100 | 107.4 ± 3.3 | 108.3 ± 1.1 | 99.2 ± 4.1 | 107.5 ± 2.4 | 108.5 ± 0.8 | 99.1 ± 2.9 | 109.0 ± 1.8 | 121.0 ± 3.6 | 90.1 ± 1.3 |

# ^a^ Each control is one of the commercially available kits described in the Experimental section.

# ^b^ Recovery (%) of NAPDH = Measured amount / Expected amount x 100.
