# Supplementary material for: Rapid and sensitive detection of NADPH via mBFP-mediated enhancement of its fluorescence
Source: PLoS One. 2019 Feb 11;14(2):e0212061. doi: 10.1371/journal.pone.0212061 (PMC6370209; doi:10.1371/journal.pone.0212061)
Supplement: S3 Table — a Mean of three repetitions ± standard deviation of the mean. (DOC) [file pone.0212061.s008.doc]

# S3 Table. Fluorescence levels of mBFP-NADPH complexes in solutions with different pH values

|  | pH | | | |
| --- | --- | --- | --- | --- |
| NADPH (pmol) | 6.0 | 7.5 | 9.0 | 10.5 |
| 5 | 19.3 ± 1.5^a^ | 41 ± 2.6 | 37.7 ± 10 | 26.3 ± 8.5 |
| 10 | 44.7 ± 8.6 | 69.7 ± 4.7 | 64.7 ± 12.9 | 54 ± 6 |
| 20 | 111.3 ± 8.5 | 141 ± 3.6 | 142.3 ± 14.6 | 97.7 ± 9.5 |
| 30 | 164.3 ± 14 | 239.3 ± 7.1 | 197 ± 17.1 | 139.7 ± 10 |
| 50 | 270.7 ± 22 | 399 ± 13.5 | 318 ± 20.7 | 214.7 ± 14 |
| 70 | 431.3 ± 11.1 | 574.3 ± 8.6 | 481.3 ± 17.4 | 318 ± 15 |
| 100 | 600 ± 16.1 | 825 ± 25.5 | 675.7 ± 35.6 | 397.7 ± 14.5 |
| 150 | 907.7 ± 26 | 1155.7 ± 28.5 | 922.3 ± 17.5 | 664.7 ± 21.5 |
| 200 | 1156 ± 23.5 | 1518.7 ± 49.2 | 1352.3 ± 31.5 | 754 ± 16.1 |
| 500 | 2720 ± 81.6 | 3910 ± 34 | 3273 ± 69.2 | 1946.3 ± 47.5 |
| 1000 | 4794.3 ± 205.5 | 6969.3 ± 197.5 | 6149 ± 68.9 | 3479.3 ± 115.5 |
| 2000 | 8634.7 ± 241 | 10733.3 ± 436 | 9944.3 ± 206.8 | 6039 ± 282 |

# ^a^ Mean of three repetitions ± standard deviation of the mean.
