# Supplementary material for: Rapid and sensitive detection of NADPH via mBFP-mediated enhancement of its fluorescence
Source: PLoS One. 2019 Feb 11;14(2):e0212061. doi: 10.1371/journal.pone.0212061 (PMC6370209; doi:10.1371/journal.pone.0212061)
Supplement: S4 Table — a Mean of three repetitions ± standard deviation of the mean. (DOC) [file pone.0212061.s009.doc]

# S4 Table. Fluorescence levels of mBFP-NADPH complexes in solutions with different temperatures

|  | Temperature (℃) | | | | |
| --- | --- | --- | --- | --- | --- |
| NADPH (pmol) | 4 | 10 | 20 | 30 | 37 |
| 5 | 55.3 ± 4.5^a^ | 37 ± 1 | 31.3 ± 1.2 | 27.7 ± 4 | 36.3 ± 11 |
| 10 | 99.7 ± 7 | 82 ± 1.7 | 62 ± 5.6 | 79.3 ± 4.2 | 39 ± 2 |
| 20 | 185 ± 3.6 | 169.3 ± 11.7 | 136 ± 14 | 129.7 ± 7.1 | 102.3 ± 10.3 |
| 30 | 266 ± 7.2 | 252.3 ± 16.3 | 203.3 ± 18.5 | 186 ± 14.1 | 138.3 ± 14.4 |
| 50 | 435.7 ± 15 | 403 ± 12 | 323.7 ± 13.5 | 313.3 ± 6.5 | 264.7 ± 25.5 |
| 70 | 598.7 ± 13.5 | 568 ± 14.5 | 467.7 ± 6.4 | 420.3 ± 9.7 | 314.3 ± 36.7 |
| 100 | 896.3 ± 27 | 846.7 ± 39.5 | 688 ± 27.6 | 624.7 ± 28.5 | 492 ± 15 |
| 150 | 1260.3 ± 31.1 | 1238.3 ± 51.2 | 1002.7 ± 49.7 | 821 ± 20.7 | 633 ± 19.1 |
| 200 | 1679.3 ± 62.7 | 1612.3 ± 86.6 | 1335.3 ± 67.9 | 1113.3 ± 33.7 | 842.3 ± 24.4 |
| 500 | 4142 ± 72 | 3975.3 ± 39.6 | 3275.3 ± 84 | 2651.7 ± 73.5 | 2037.3 ± 44.5 |
| 1000 | 7468 ± 143.7 | 7267.7 ± 98 | 5581.3 ± 141.5 | 5131.7 ± 152.5 | 3540 ± 85.5 |
| 2000 | 12384 ± 347 | 12210.7 ± 349 | 8809 ± 215.5 | 7787 ± 198.5 | 4624 ± 124.8 |

# ^a^ Mean of three repetitions ± standard deviation of the mean.
