# Supplementary material for: Rapid and sensitive detection of NADPH via mBFP-mediated enhancement of its fluorescence
Source: PLoS One. 2019 Feb 11;14(2):e0212061. doi: 10.1371/journal.pone.0212061 (PMC6370209; doi:10.1371/journal.pone.0212061)
Supplement: S5 Table — a Mean of three repetitions ± standard deviation of the mean. (DOC) [file pone.0212061.s010.doc]

# S5 Table. Effect of non-NADPH nicotinamide cofactors on the fluorescence level of mBFP-NADPH complexes.

| Ratio | NADP^+^/NADPH | NADH/NADPH | NAD^+^/NADPH |
| --- | --- | --- | --- |
| 0.4 | 92.3 ± 4.2^a^ | 100.6 ± 4.8 | 98.2 ± 2.2 |
| 1.0 | 90.4 ± 2 | 98.5 ± 1.1 | 93.9 ± 3.5 |
| 2.0 | 84.1 ± 5.7 | 98.1 ± 4.4 | 96.6 ± 2.4 |
| 4.0 | 74.3 ± 7.1 | 88.2 ± 3.2 | 96.6 ± 4.8 |
| 6.0 | 69.9 ± 6.7 | 86.6 ± 6 | 94.6 ± 3.5 |
| 8.0 | 64.4 ± 9.6 | 82.1 ± 2.3 | 94.5 ± 2.5 |
| 10.0 | 57.7 ± 20.5 | 83.2 ± 1.3 | 91.5 ± 3.4 |

# ^a^ Mean of three repetitions ± standard deviation of the mean.
