# Supplementary material for: Rapid and sensitive detection of NADPH via mBFP-mediated enhancement of its fluorescence
Source: PLoS One. 2019 Feb 11;14(2):e0212061. doi: 10.1371/journal.pone.0212061 (PMC6370209; doi:10.1371/journal.pone.0212061)
Supplement: S6 Table — a Mean of three repetitions ± standard deviation of the mean. (DOC) [file pone.0212061.s011.doc]

# S6 Table. Dependency of fluorescence signal on the quantity of NADPH upon the addition of different concentrations of mBFP

|  | mBFP (μM) | | | | |
| --- | --- | --- | --- | --- | --- |
| NADPH (pmol) | 0 | 0.1 | 1 | 5 | 10 |
| 5 | 5 ± 1^a^ | 24.3 ± 5.7 | 42.7 ± 7 | 36 ± 10 | 60.3 ± 10 |
| 10 | 10.7 ± 1.5 | 50.7 ± 9.5 | 83.7 ± 16 | 91.7 ± 14.5 | 114 ± 10.5 |
| 20 | 19.3 ± 2.5 | 131 ± 10.1 | 255 ± 7.5 | 256.7 ± 8.6 | 230.7 ± 18.8 |
| 30 | 28.7 ± 2.1 | 172 ± 17.1 | 320.7 ± 11.2 | 346.7 ± 22.5 | 341.3 ± 17.6 |
| 50 | 45 ± 2.6 | 201.7 ± 20 | 388.7 ± 16.6 | 467.3 ± 10 | 540.7 ± 39.3 |
| 70 | 62.7 ± 1.2 | 310 ± 9.5 | 643 ± 15.1 | 708.3 ± 28.1 | 771.3 ± 14.6 |
| 100 | 89.7 ± 1.5 | 374 ± 19 | 753.7 ± 30 | 1008.3 ± 30 | 1067.7 ± 54.4 |
| 200 | 179 ± 3 | 630 ± 11.1 | 1616.7 ± 58 | 1874.3 ± 66.6 | 2211.3 ± 70 |
| 500 | 440 ± 11.4 | 708.3 ± 43 | 1960.7 ± 83.6 | 4490.7 ± 180 | 4679.7 ± 164.1 |
| 1000 | 864 ± 8.5 | 1114 ± 50 | 2847.7 ± 70.6 | 7647.7 ± 150.5 | 8800 ± 135.2 |
| 2000 | 1764 ± 27.2 | 1935.3 ± | 3492.7 ± 249.5 | 13163 ± 356.3 | 15833 ± 527.4 |

# ^a^ Mean of three repetitions ± standard deviation of the mean.
