# Supplementary material for: Rapid and sensitive detection of NADPH via mBFP-mediated enhancement of its fluorescence
Source: PLoS One. 2019 Feb 11;14(2):e0212061. doi: 10.1371/journal.pone.0212061 (PMC6370209; doi:10.1371/journal.pone.0212061)
Supplement: S7 Table — a Mean of three repetitions ± standard deviation of the mean. (DOC) [file pone.0212061.s012.doc]

# S7 Table. Stability of fluorescent signals after the addition of mBFP to solutions containing different amounts of NADPH.

|  | NADPH (pmol) | | | |
| --- | --- | --- | --- | --- |
| Time (min) | 100 | 250 | 500 | 1000 |
| 0 | 1199 ± 32.1^a^ | 2646.3 ± 64.5 | 4917.3 ± 138.5 | 7619.3 ± 133.7 |
| 0.5 | 1112.7 ± 60 | 2586.3 ± 59.3 | 4735 ± 129 | 7575.3 ± 107.5 |
| 1 | 1170.7 ± 30.7 | 2536 ± 36.4 | 4596.3 ± 65.5 | 7469 ± 105 |
| 5 | 1084.7 ± 12.9 | 2483.3 ± 18.7 | 4247.7 ± 105.8 | 7177.3 ± 93.6 |
| 10 | 813.3 ± 12.7 | 1920.3 ± 76.2 | 3631.3 ± 60.5 | 6394 ± 117.8 |
| 30 | 572 ± 11.8 | 1478.7 ± 44.2 | 2683 ± 74.9 | 4741.3 ± 93.2 |
| 60 | 379.7 ± 14.4 | 1027.3 ± 42.7 | 1983.3 ± 82 | 3419.7 ± 93.8 |

# ^a^ Mean of three repetitions ± standard deviation of the mean.
