# Supplementary material for: Rapid and sensitive detection of NADPH via mBFP-mediated enhancement of its fluorescence
Source: PLoS One. 2019 Feb 11;14(2):e0212061. doi: 10.1371/journal.pone.0212061 (PMC6370209; doi:10.1371/journal.pone.0212061)
Supplement: S8 Table — a Mean of three repetitions ± standard deviation of the mean. (DOC) [file pone.0212061.s013.doc]

# S8 Table. Kinetic profiles of NADPH production by G6PDH in a microplate format.

|  | G6PDH (mU) | | | | |
| --- | --- | --- | --- | --- | --- |
| Time (min) | 0.05 | 0.1 | 0.25 | 0.5 | 1 |
| 1 | 172.7 ± 15.2^a^ | 218.7 ± 12.1 | 1299.7 ± 75.3 | 2577 ± 21.9 | 4971 ± 73.6 |
| 3 | 1075 ± 19 | 1897 ± 133.1 | 4552.7 ± 122.5 | 7899 ± 167.9 | 15071 ± 239.5 |
| 5 | 1562.7 ± 6.4 | 3485.3 ± 66 | 6832.7 ± 100.8 | 11089.3 ± 270.1 | 19540 ± 425.6 |
| 7 | 1988.3 ± 52.6 | 5505.7 ± 66.6 | 9628 ± 313.6 | 13588.3 ± 233 | 22516.7 ± 553.9 |
| 10 | 2547 ± 11.8 | 7067 ± 89 | 12630 ± 485.1 | 16453.3 ± 212.5 | 24104.7 ± 495 |

# ^a^ Mean of three repetitions ± standard deviation of the mean.
